# Extracellular Succinate Modulates Neuroimmune Responses in a Murine Microglial Cell Line

**DOI:** 10.3390/biom16030407

**Published:** 2026-03-10

**Authors:** Samantha C. Y. Yudin, Kimberly Day, Erica Y. Scott, Meha N. Patel, Hashim Islam, Andis Klegeris

**Affiliations:** 1 Department of Biology, Faculty of Science, University of British Columbia Okanagan Campus, Kelowna, BC V1V 1V7, Canada; 2 School of Health and Exercise Sciences, University of British Columbia Okanagan Campus, Kelowna, BC V1V 1V7, Canada

**Keywords:** damage-associated molecular patterns, glia, immunometabolism, intercellular signaling, mitochondrial respiration, neurodegenerative diseases, neuroinflammation

## Abstract

Neuroinflammation mediated by reactive microglia, the immune cells of the brain, contributes to numerous neuropathologies. Damage-associated molecular patterns (DAMPs), released from stressed or damaged cells, are implicated in neuroinflammation. Succinate, a tricarboxylic acid cycle intermediate, can accumulate intracellularly and be released into the extracellular space where it may function as a DAMP-like molecule. However, its specific roles in central nervous system (CNS) neuroimmune responses, particularly when acting extracellularly, remain largely unexplored. This study utilizes cell membrane-impermeable disodium succinate to model extracellular action and cell-permeable diethyl succinate to assess the intracellular activity of this metabolite in cell culture models. We demonstrate that extracellular disodium succinate significantly reduces the secretion of pro-inflammatory cytokines tumor necrosis factor-α (TNF) and interleukin (IL)-6, and lowers neurotoxic and phagocytic activities of immune-stimulated BV-2 murine microglia. It also rescues lipopolysaccharide (LPS)-induced decreases in mitochondrial respiration in human peripheral blood mononuclear cells (PBMCs) used as microglia models, which correlates with its actions on phagocytosis. In contrast, while intracellular diethyl succinate reduces TNF and IL-6 secretion, it does not reduce BV-2 microglia toxicity towards murine NSC-34 neuronal cells, indicating location-dependent effects. These results support extracellular succinate as a novel CNS DAMP with a predominantly anti-inflammatory action on microglia.

## 1. Introduction

Alzheimer’s disease (AD) is the leading cause of dementia. This neurodegenerative disorder affects 12% of women and 9% of men over the age of 65 [1]. Historically, the formation of extracellular plaques containing deposited amyloid-β (Aβ) proteins and intracellular neurofibrillary tangles of tau proteins were recognized as the main neuropathological hallmarks of AD [2,3]. However, accumulating evidence suggests that adverse activation of microglia, the resident immune cells of the brain, plays a key role in mediating the neuroinflammation and neuronal death observed in AD. Typically, in a healthy brain, microglia participate in tissue maintenance, injury repair, and response to pathogens [2]. Upon pathological stimulation, this homeostatic behaviour of microglia is lost, and they acquire pro-inflammatory reactive phenotypes. Studies have demonstrated that reactive microglia in neurodegenerative diseases are characterized by dysregulated immune functions including upregulated secretion of inflammatory mediators, such as nitric oxide (NO), tumor necrosis factor-α (TNF), and interleukin (IL)-6, as well as excessive engulfment of neuronal synapses through phagocytosis [4,5,6,7]. Thus, understanding this microglial reactivity and the regulation of their immune responses may provide additional insights into the pathogenesis of AD and lead to identification of therapeutic targets for treating neuroimmune disorders.

Damage-associated molecular patterns (DAMPs) are endogenous molecules that, upon release into the extracellular space following cellular stress or injury, function as potent immunostimulatory signals [8,9]. Under homeostatic conditions, they are sequestered within intracellular compartments—such as the mitochondria, nucleus, cytosol, or endoplasmic reticulum—where they carry out specific molecular functions. After entering the extracellular space, these molecules are recognized by pattern recognition receptors (PRRs) of microglia, potentially contributing to the pathophysiology of neurodegenerative diseases such as AD [10]. As such, identification of novel DAMPs can improve our understanding of central nervous system (CNS) disorders and uncover potential biomarkers and therapeutic targets.

Succinate has recently emerged as a strong candidate for serving as a DAMP-like signaling molecule within the CNS. This dicarboxylic acid primarily functions as a critical intermediate in the tricarboxylic acid (TCA) cycle, where it aids in cell metabolism and the production of adenosine triphosphate (ATP). In the TCA cycle, succinyl-coenzyme A (CoA) synthetase catalyzes the synthesis of succinate from succinyl-CoA. Succinate is then converted to fumarate by the enzyme succinate dehydrogenase (SDH) [11]. In immune cells, this series of reactions can become interrupted in several ways, leading to succinate accumulation. For example, macrophages and dendritic cells in hypoxic inflammatory microenvironments can undergo a metabolic shift toward glycolysis, which results in inhibition of SDH and consequently prevents the conversion of succinate to fumarate [12]. In the CNS pathologies, transition of microglia into a reactive, pro-inflammatory state and a subsequent increase in glycolysis causes intracellular succinate accumulation [13]. Additionally, the γ-aminobutyric acid (GABA) shunt, triggered by stressors such as hypoperfusion and mitochondrial dysfunction, can produce excess succinate in AD brains [14]. Upon accumulation, succinate can be transported across the mitochondrial inner membrane by solute carrier family 25 member 10 (SLC25A10), effectively equilibrating mitochondrial and cytosolic concentrations of succinate [15]. While succinate may remain inside cells, it can also be released into the extracellular space through the plasma-membrane dicarboxylic transporter, solute carrier family 13 member 3 (SLC13A3) [16,17]. Once extracellular, succinate modulates macrophage TNF, IL-1β, and nitric oxide synthase 2 (Nos2), and has therefore been considered a DAMP in peripheral tissues [16]; it may have similar extracellular activity in the CNS. 

Compared to other TCA intermediates, succinate is of additional interest, as it can function in roles unrelated to energy production as a paracrine and endocrine signaling molecule. Evidence obtained in the peripheral tissues indicates that succinate regulates the functions of immune cells. In bone marrow-derived macrophages (BMDMs), succinate acting intracellularly suppresses lipopolysaccharide (LPS)-induced secretion of TNF and NO [18]. Additionally, extracellular succinate can act as an endogenous ligand of the succinate receptor (SUCNR1) expressed on the cell surface of macrophages, dendritic cells, and microglia [19]. Deficiency of SUCNR1 promotes a pro-inflammatory phenotype and impaired glucose tolerance in murine myeloid cells, highlighting the potential role of succinate-SUCNR1 signaling in limiting an inflammatory response [16].

The effects of succinate on CNS glia immunometabolic responses, especially when acting extracellularly, have been investigated only to a limited extent. Pre-treatment of immune-stimulated primary murine microglia with a cell-permeable succinate derivative has been shown to decrease microglia polarization to a pro-inflammatory phenotype and their reactive oxygen intermediates (ROS) production [20]. Additionally, Kirova et al. [21] find that intraperitoneal injection of the ethylmethylhydroxypyridine derivative of succinate, capable of crossing the blood–brain barrier (BBB), lowers pro-inflammatory cytokine expression in the brains of 18-month-old mice. However, it remains unclear whether this effect is due to intracellular or extracellular action of succinate. Given the limited studies in the CNS that explicitly control for succinate localization, further research is needed to determine whether extracellular and intracellular succinate have distinct effects on microglial immune functions. We therefore hypothesized that extracellular succinate acts as a DAMP-like molecule in the CNS, selectively modulating microglial immune functions and metabolism in a manner distinct from intracellular succinate. While intracellular effects of succinate have been studied in peripheral immune cells and microglia, its extracellular signaling in the CNS remains largely unexplored; this study is the first to link extracellular succinate to anti-inflammatory, neuroprotective, and metabolic effects in microglia in a cell location-dependent manner.

To address this knowledge gap, we used the cell membrane-impermeable disodium derivative to model succinate acting extracellularly, while diethyl succinate was employed to explore its effects when acting intracellularly [16,18,22]. Two different microglia-like cell types were used to measure the following parameters of their immune activation: secretion of NO and proinflammatory cytokines TNF and IL-6 by BV-2 murine microglia-like cells; cytotoxic action of BV-2 microglia towards NSC-34 neuronal cells; phagocytic activity of BV-2 microglia; and changes in mitochondrial respiration parameters of primary human peripheral blood mononuclear cells (PBMCs). Our findings demonstrate that succinate differentially regulates select microglial immune functions depending on its intra- versus extracellular localization.

## 2. Materials and Methods

### 2.1. Reagents and Cells

Disodium succinate, diethyl succinate, LPS (from Escherichia coli O55:B5), 3-(4,5-dimethyl-2-thiazolyl)-2,5-diphenyl-2H-tetrazolium bromide (MTT), bisbenzimide trihydrochloride (Hoechst 33258), poly-D-lysine, carboxylate-modified polystyrene fluorescent yellow-green latex beads (1.0 µm mean particle size), trypan blue solution (0.4%), malate, pyruvate, glutamate, digitonin, adenosine diphosphate (ADP), succinate, carbonyl cyanide m-chlorophenyl hydrazone (CCCP), and N,N-dimethylformamide were purchased from Sigma Aldrich (Oakville, ON, Canada). Recombinant murine interferon (IFN)-γ, enzyme-linked immunosorbent assay (ELISA) development kits used to measure concentrations of murine TNF and IL-6, calf bovine serum (CBS), fetal bovine serum (FBS), Dulbecco’s modified Eagle medium/Ham’s F-12 nutrient mixture (DMEM-F12), penicillin/streptomycin/amphotericin B stock solution, 0.25% trypsin with ethylenediaminetetraacetic acid (EDTA), and all other reagents were purchased from ThermoFisher Scientific (Ottawa, ON, Canada). 

BV-2 murine microglial cells, provided by Dr. G. Garden (University of Washington, Seattle, WA, USA), and NSC-34 murine neuronal cells, acquired from Dr. A. Milnerwood (Brain Research Centre, University of British Columbia (UBC), Vancouver, BC, Canada), were cultured in T75 flasks. Cell lines were maintained in DMEM-F12 medium supplemented with 10% heat-inactivated CBS, 100 U/mL penicillin, 100 µg/mL streptomycin, and 500 ng/mL amphotericin B. Cultures were incubated at 37 °C in a humidified atmosphere of 95% air and 5% CO_2_.

To obtain PBMCs, blood samples were obtained following an overnight fast (8–12 h) from five healthy adults (3 males, 2 females; age = 25 ± 3 [mean ± standard deviation] years; body mass index = 25.1 ± 2.3 kg/m^2^) by standard venipuncture of an antecubital vein using a 21-gauge butterfly needle (Becton Dickinson, Franklin Lakes, NJ, USA). Informed consent was obtained from all participants prior to sample collection and the protocol (#H25-01094) was approved by the UBC Clinical Research Ethics Board. Blood was collected into EDTA-coated vacutainer tubes (Becton Dickinson) and processed within 15 min of collection. PBMCs were isolated from whole blood by gradient density centrifugation using Histopaque 1077 (Sigma Aldrich, Oakville, ON, Canada). Whole blood was layered onto Histopaque in sterile LeucoSep tubes (Greiner Bio-One, Monroe, NC, USA) and centrifuged for 20 min at 800× *g* at room temperature without braking. The PBMC layer was recovered and washed twice with warm sterile phosphate-buffered saline (PBS) (400× *g* for 10 min at room temperature). Isolated PBMCs were resuspended in RPMI medium (Corning, Corning, NY, USA) supplemented with 5 mM glucose, 10% FBS and 50 µg/mL penicillin-streptomycin, counted using an automated cell counter (Countess, ThermoFisher Scientific, Waltham, MA, USA), and seeded in a 12-well culture plate at a density of 2 × 10^6^ live cells per replicate well in 2 mL culture media. PBMCs were then treated with disodium succinate (24 mM), LPS (400 ng/mL), or both, for 24 h. Following stimulation, cells were gently removed using a cell lifter, pelleted (400× *g* for 10 min at room temperature), resuspended in mitochondrial respiratory buffer (MiR05, Oroboros Instruments, Innsbruck, Austria), and used for high-resolution respirometry. 

### 2.2. Measurement of Cytokines, NO, and BV-2 Microglia-Mediated Neurotoxicity

BV-2 cells were seeded into 24-well plates at a density of 1 × 10^5^ cells/mL in 1 mL of DMEM-F12 containing 5% CBS and antibiotics. After a 24 h incubation period to allow for cell adherence, the medium was replaced, and cells were exposed to varying concentrations of disodium succinate, diethyl succinate, or their vehicle (distilled H_2_O). Cells were treated 3 h later with 400 ng/mL LPS, 30 ng/mL IFN-γ, a combination of the two stimulants, or with PBS vehicle as a control. Cell-free supernatants were collected following another 24 h incubation period and BV-2 cell viability measured by the MTT assay. Nitrite, a stable breakdown product of NO, was quantified as described previously [23]. 50 μL of Griess reagent (1% sulfanilamide, 2.5% phosphoric acid, 0.1% N-(1-naphthyl)ethylenediamine dihydrochloride in distilled water) were added to 50 μL of cell-free supernatants. Optical densities at 570 nm were measured using a microplate reader (Molecular Devices, San Jose, CA, USA) and nitrite concentrations were interpolated from a standard curve prepared with defined concentrations of nitrite in cell culture medium. Concentrations of TNF and IL-6 were measured in cell-free supernatants using ELISA development kits according to the manufacturer’s protocol (ThermoFisher Scientific). 

The effects of disodium succinate and diethyl succinate on microglia-mediated neurotoxicity were assessed using a previously published protocol with minor modifications [24]. 300 μL cell-free supernatants collected from treated BV-2 cells plus 100 μL of fresh DMEM-F12 containing antibiotics and 10% CBS were transferred to separate wells containing NSC-34 neuronal cells that had been seeded 24 h earlier at 4 × 10^5^ cells/mL in 400 μL of DMEM-F12 containing antibiotics and 5% CBS. After 72 h incubation, NSC-34 neuronal cell viability was measured by the MTT assay.

### 2.3. Phagocytic Activity of BV-2 Microglial Cells

Phagocytosis of fluorescent latex beads by microglial cells was quantified as previously described with minor modifications [25,26]. Four-chambered glass-bottom dishes (Greiner Bio-One) were coated with 500 µL of 50 mg/mL poly-D-lysine for 1 h, followed by three washes with PBS. BV-2 cells were seeded at a density of 5 × 10^4^ cells/mL in 500 µL of DMEM-F12 supplemented with 5% CBS and antibiotics. After a 24 h incubation period to allow for adherence, cells were treated with 24 mM disodium succinate or distilled H_2_O vehicle solution. Following a 3 h treatment, cells were exposed to 400 ng/mL LPS or its PBS vehicle solution and incubated for an additional 24 h period. 2 µL of fluorescent latex beads were added to each well for 1 h followed by 100 µL of 0.4% trypan blue solution for 1 min. Cells were washed with 500 µL of warm PBS, then fixed in 500 µL of 4% paraformaldehyde at room temperature for 5 min. Cell nuclei were stained by adding 5 µL of 200 µg/mL bisbenzimide to 500 µL of PBS before cells were imaged at 40× magnification using a Zeiss Axio Observer Z1 (Carl Zeiss AG, Oberkochen, Germany) inverted widefield fluorescence microscope and ZEN 2.0 software. Fluorescence intensities were quantified using Fiji/ImageJ software (version 1.53, National Institutes of Health, Bethesda, MD, USA), as previously described [25]. Measurements were performed by an investigator blinded to the experimental conditions. Corrected total cell fluorescence (CTCF) for each cell was calculated by measuring the total fluorescence within the cell area and subtracting background fluorescence from cell-free regions, accounting for differences in cell size and non-specific signal.

### 2.4. Digital Polymerase Chain Reaction (PCR) Analysis of Sucnr1 Expression in BV-2 Microglia

Complementary DNA (cDNA) was synthesized using the SuperScript^TM^ IV CellsDirect^TM^ cDNA Synthesis Kit (11750150, Invitrogen, Carlsbad, CA, USA) according to the manufacturer’s instructions. BV-2 murine microglial cells were treated with IFN-γ (30 ng/mL) and LPS (400 ng/mL) for 6 h or 12 h, or with PBS vehicle as a control for 6 h. The resulting pellet, consisting of 3 × 10^6^ cells, was resuspended in 10 μL PBS, and 5 μL of this resuspension was used for cDNA synthesis with the SuperScript^TM^ IV CellsDirect^TM^ kit. Gene expression was quantified using the QuantStudio Absolute Q Digital PCR System, with the Absolute Q^TM^ Universal DNA Digital PCR Master Mix, 5× (A72710, ThermoFisher Scientific), *Sucnr1* primers (4331182, assay ID: Mm02620543_s1, ThermoFisher Scientific), and beta-actin (*Actb*) primers (4331182, assay ID: Mm00607939_s1, ThermoFisher Scientific). For each digital PCR assay, 1 μL of a 1:10 dilution of the SuperScript^TM^ IV CellsDirect^TM^ cDNA synthesis product was used. Negative controls included no-template controls and no-reverse transcriptase (RT) controls provided by SuperScript^TM^ IV CellsDirect^TM^ cDNA Synthesis kit. Raw *Sucnr1* and *Actb* expression data are shown in Supplementary Figure S4.

### 2.5. High Resolution Respirometry of PBMCs

Mitochondrial respiration of PBMCs was determined using high-resolution respirometry (Oxygraph-2K, Oroboros Instruments, Innsbruck, Austria). Standard air calibration was performed on each experimental day according to manufacturer instructions. Following determination of cell counts using an automated cell counter (Countess, ThermoFisher), 4 × 10^6^ live PBMCs were added to each 0.5 mL chamber containing MiR05 maintained at 37 °C with constant stirring (750 rpm). Routine (basal) respiration was determined in intact PBMCs prior to cell permeabilization using digitonin (5 µg/mL). Complex I-supported oxidative phosphorylation (OXPHOS) was determined in the presence of malate (2 mM), pyruvate (5 mM), glutamate (10 mM) and ADP (2.5 mM). Complex I- and II-supported OXPHOS was then determined after adding succinate (10 mM), followed by titrations with the uncoupler CCCP (0.5 μM steps) to determine maximal electron transfer capacity (uncoupled respiration). Data were recorded and analyzed in DatLab (version 7.4, Oroboros Instruments). Oxygen consumption is expressed in pmol/sec/10^6^ cells. 

### 2.6. Measurement of Cell Viability

Cell viability was monitored by an assay that measured the reduction in MTT to insoluble purple formazan crystals by viable cells [27]. A previously described procedure was used with minor modifications [28]. Cell cultures used in this study were exposed to MTT (0.5 mg/mL) for 1 h, followed by the addition of a solubilizing solution (20% *w*/*v* sodium lauryl sulfate and 50% *v*/*v* N,N-dimethylformamide in water) in a 1:1 ratio with well volume. The crystals were dissolved by using an orbital plate shaker for 1 h. Optical density was measured at 570 nm using a microplate reader (Molecular Devices, San Jose, CA, USA). The data were normalized using values obtained from cells incubated in fresh growth medium only.

### 2.7. Statistical Analyses

Data were analyzed using the randomized block design one-way analysis of variance (ANOVA) followed by either Dunnett’s or Tukey’s post hoc test. Statistical significance was established at *p* < 0.05. Independent experiments were performed on separate days and data are presented as means ± standard error of the mean (SEM). Statistical analyses and graphing were completed using Prism software (version 10.1.0, GraphPad Software, La Jolla, CA, USA). For digital PCR experiments, gene expression data were log-transformed for visualization purposes only; all statistical analyses were performed on untransformed data. Because these data did not meet the assumptions of normality, non-parametric statistical analyses were applied. Comparisons between two groups were performed using the Wilcoxon rank-sum exact test.

## 3. Results

### 3.1. Effects of Disodium Succinate on the Secretion of TNF, IL-6, and NO by BV-2 Murine Microglia

**Figure 1:**
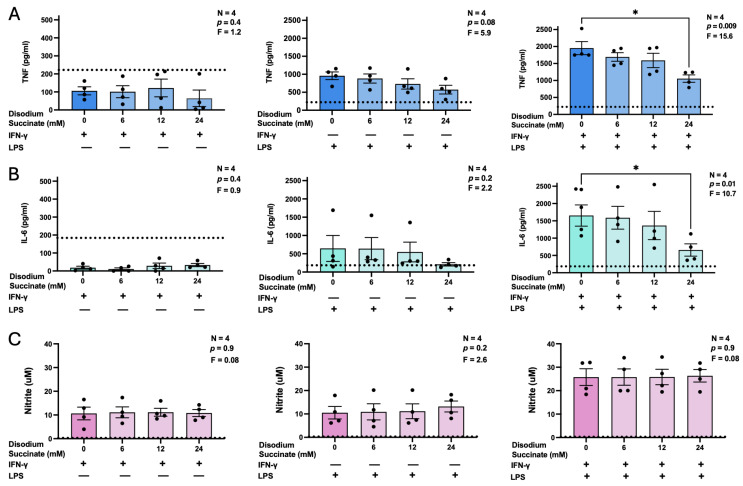
The effects of disodium succinate on the secretion of TNF (**A**), IL-6 (**B**), and NO (**C**) by BV-2 murine microglia. Cells were treated with disodium succinate for 3 h (0–24 mM) prior to stimulation with IFN-γ (30 ng/mL), LPS (400 ng/mL), or IFN-γ (30 ng/mL) plus LPS (400 ng/mL). Following a 24 h incubation period, concentrations of cytokines and nitrite in cell-free supernatants were measured by ELISAs and the Griess assay, respectively. Data from four independent experiments completed on different days are shown as means ± SEM. The displayed *p* and F values are calculated using randomized block one-way ANOVA, and the detection limit of each assay is indicated by a dotted line. * *p* < 0.05 according to Dunnett’s post hoc test.

Although succinate may function as a DAMP of the CNS, it is unclear whether its extracellular actions have overall pro- or anti-inflammatory effects on glial cells [29]; it has been shown, however, to enhance TNF secretion following LPS and IFN-γ stimulation of human iPSC-derived microglia [30]. Furthermore, it remains unclear whether the immunomodulatory effects of succinate are mediated by its intracellular or extracellular actions; therefore, we investigated whether disodium succinate—a membrane-impermeable derivative of succinate—modulates the inflammatory response of BV-2 murine microglia. Specifically, we measured the secretion of three pro-inflammatory and potentially neurotoxic molecules: TNF, IL-6, and NO. We selected LPS, IFN-γ, and their combination as immune stimulants because they have been shown to engage distinct microglia receptors and intracellular signaling pathways, as well as to trigger the release of the above inflammatory mediators by BV-2 microglia specifically [4]. First, we confirmed that unstimulated BV-2 cells do not secrete detectable levels of TNF, IL-6, or NO, and demonstrated that disodium succinate at non-toxic concentrations of up to 24 mM did not induce the release of any of these three molecules (Supplementary Figure S1). Next, we observed that pre-treatment with 24 mM disodium succinate significantly reduced TNF (Figure 1A) and IL-6 (Figure 1B) secretion by BV-2 cells stimulated with a combination of LPS and IFN-γ. Although a trend of decreased cytokine levels was observed in supernatants from cells stimulated with LPS alone, this effect did not reach statistical significance. Neither TNF nor IL-6 was detected in cell cultures treated with IFN-γ alone or in combination with disodium succinate (Figure 1A,B). Furthermore, disodium succinate had no effect on BV-2 microglia NO production under any of the stimulatory conditions used in this study (Figure 1C).

### 3.2. Effects of Disodium Succinate on Cytotoxicity of BV-2 Murine Microglia Towards NSC-34 Neuronal Cells

Cytotoxic secretions of immune-stimulated microglia can be directly harmful to neurons, a phenomenon that could be relevant to pathophysiology of numerous neurodegenerative disorders [5,31,32]. To the best of our knowledge, it is unknown whether extracellular succinate can modulate microglia-induced neuronal death. To address this knowledge gap, we conducted an in vitro assay in which supernatants from BV-2 cells, first treated with disodium succinate and then immune stimulated as described in the previous experiment, were transferred to cultured NSC-34 neuronal cells. Neuronal viability was assessed 72 h later as a measure of microglia-induced cytotoxicity.

**Figure 2:**
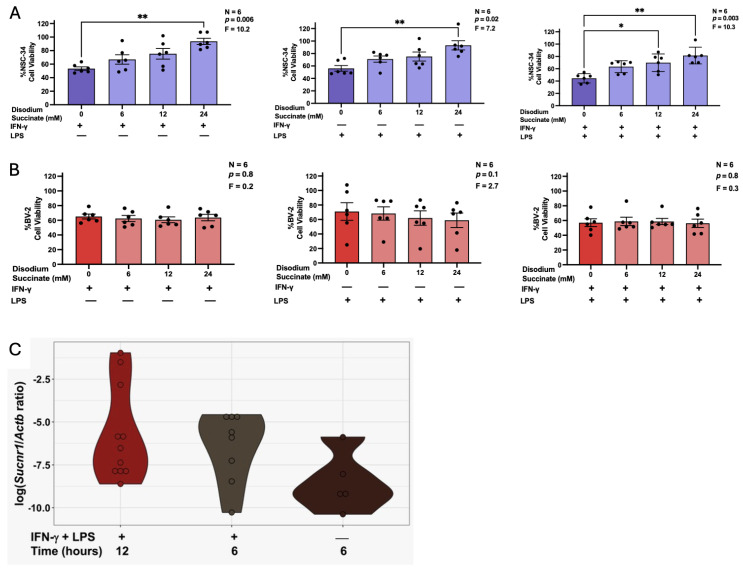
The effects of disodium succinate on BV-2 murine microglia toxicity towards NSC-34 murine neuron-like cells (**A**) and viability of stimulated BV-2 murine microglia (**B**). A violin plot showing the ratio of *Sucnr1* gene expression to *Actb* housekeeping gene expression in unstimulated and stimulated BV-2 murine microglia (**C**). (**A**,**B**): BV-2 cells were treated with disodium succinate (0–24 mM) for 3 h prior to stimulation with IFN-γ (30 ng/mL), LPS (400 ng/mL), or IFN-γ (30 ng/mL) plus LPS (400 ng/mL). Following a 24 h incubation period, supernatants from BV-2 cells were transferred onto NSC-34 cells and the viability of BV-2 cells was measured using the MTT assay (**B**). 72 h later, the viability of NSC-34 cells was quantified using the MTT assay (**A**). Data from six independent experiments completed on different days are shown as means ± SEM. The displayed *p* and F values are calculated using randomized block one-way ANOVA. * *p* < 0.05 and ** *p* < 0.01 according to Dunnett’s post hoc test. (**C**): BV-2 cells were stimulated with IFN-γ (30 ng/mL) and LPS (400 ng/mL) for 6 or 12 h, or left unstimulated for 6 h. Gene expression ratios were calculated from concentrations obtained using the Applied Biosystems QuantStudio Absolute Q Digital PCR System and log transformed for visualization. *Sucnr1* expression was significantly higher in cells stimulated for 12 h with IFN-γ and LPS (n = 11) compared with unstimulated controls (n = 5) (Wilcoxon rank-sum exact test, *p* = 0.016), whereas stimulation for 6 h (n = 8) did not produce a significant increase relative to controls (Wilcoxon rank-sum exact test, *p* = 0.09).

Consistent with previous studies [4], supernatants from BV-2 cells stimulated with LPS, IFN-γ, or their combination, significantly reduced NSC-34 viability compared to control cells incubated in fresh cell culture medium. Figure 2A demonstrates that pre-treatment of BV-2 cells with 24 mM disodium succinate had a protective effect across all three stimulatory conditions as evidenced by the increased NSC-34 viability. In the LPS plus IFN-γ-stimulated BV-2 cell cultures, disodium succinate at a lower 12 mM concentration also significantly reduced cytotoxicity of supernatants. Importantly, the inhibition of microglia-mediated neurotoxicity was not due to lowered BV-2 cell viability in the presence of disodium succinate (Figure 2B). LPS, IFN-γ, or their combination, as well as disodium succinate and diethyl succinate at the concentrations used to treat BV-2 microglia, had no direct effect on NSC-34 cell viability (Supplementary Figure S3). Therefore, any residual amounts of these stimulants or succinate transferred with BV-2 cell supernatants to neuronal cultures are unlikely to have affected NSC-34 cell viability.

We also confirmed expression of the receptor for extracellular succinate in BV-2 murine microglia. Figure 2C illustrates that *Sucnr1* expression (normalized to the *Actb* housekeeping gene) differed across stimulation conditions, with the strongest induction observed following 12 h of IFN-γ plus LPS treatment. *Sucnr1* expression in these samples was significantly higher than in unstimulated controls, whereas stimulation for 6 h caused only a modest upregulation that did not reach statistical significance compared to unstimulated cells. Nevertheless, these data demonstrate that BV-2 microglia express *Sucnr1* and that IFN-γ plus LPS stimulation drives a variable but distinct upregulation of this gene.

### 3.3. Effects of Diethyl Succinate on Secretion of TNF and IL-6 by BV-2 Murine Microglia and Their Toxicity Towards NSC-34 Neuronal Cells

Few studies have directly compared the effects of succinate localized to the outside of the cell membrane compared to when it acts within the cytosol. To investigate whether modulation of microglial immune responses and neurotoxicity by succinate is location-dependent, we treated BV-2 microglial cells with a cell membrane–permeable diethyl derivative of succinate and measured cellular responses that had been significantly affected by disodium succinate in previous experiments (see Figure 1A,B and Figure 2A). Using the same experimental approach as for disodium succinate, we measured TNF and IL-6 secretion and assessed the neurotoxicity of immune-stimulated BV-2 cells in the presence or absence of diethyl succinate.

**Figure 3:**
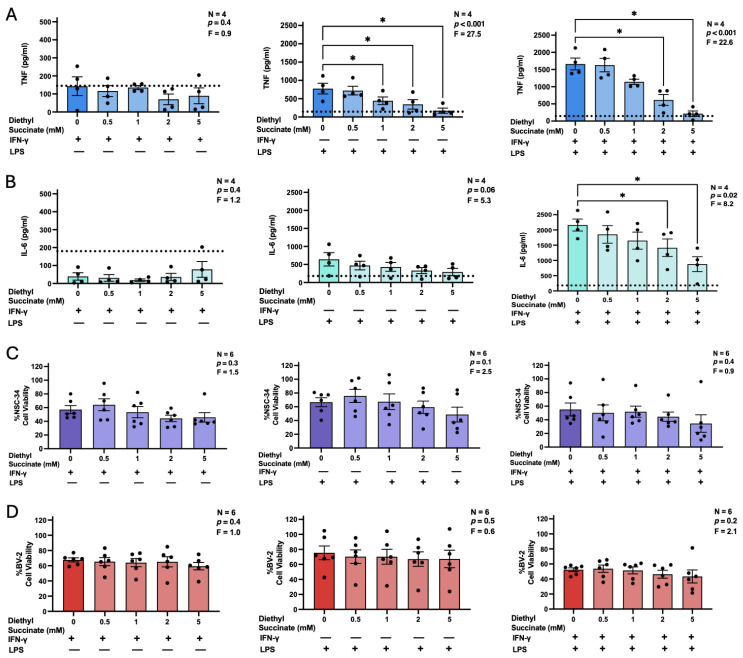
The effects of diethyl succinate on the secretion of TNF (**A**) and IL-6 (**B**) by BV-2 murine microglia, BV-2 murine microglia toxicity towards NSC-34 murine neuron-like cells (**C**), and viability of stimulated BV-2 cells (**D**). BV-2 cells were treated with diethyl succinate (0–5 mM) for 3 h prior to stimulation with IFN-γ (30 ng/mL), LPS (400 ng/mL), or IFN-γ (30 ng/mL) plus LPS (400 ng/mL). Following a 24 h incubation period, concentrations of IL-6 in cell-free supernatants were measured by an ELISA (**A**,**B**). Supernatants from BV-2 cells were also transferred onto NSC-34 cells and the viability of BV-2 cells was measured using the MTT assay (**D**). 72 h later, the viability of NSC-34 cells was quantified using the MTT assay (**C**). Data from four (**A**,**B**) or six (**C**,**D**) independent experiments completed on different days are shown as means ± SEM. The displayed *p* and F values are calculated using randomized block one-way ANOVA, and the detection limit of the ELISA is indicated by a dotted line. * *p* < 0.05 according to Dunnett’s post hoc test.

Similarly to its disodium derivative, diethyl succinate at non-toxic concentrations of up to 5 mM failed to induce TNF or IL-6 secretion by BV-2 microglia on its own (Supplementary Figure S2) or in combination with IFN-γ (Figure 3A,B). Diethyl succinate at 1–5 mM significantly reduced LPS- and LPS plus IFN-γ-induced TNF secretion by BV-2 microglia (Figure 3A). Treatment of LPS plus IFN-γ-stimulated BV-2 cells with 2 or 5 mM diethyl succinate also led to a significant reduction in IL-6 secretion. A similar trend, though not statistically significant, was observed in BV-2 cells stimulated with LPS alone (Figure 3B). The inhibitory activity of diethyl succinate was not due to its direct toxicity towards BV-2 cells (Figure 3D). Despite its effect on TNF and IL-6 secretion, and in contrast to the inhibitory effect of the disodium derivative, diethyl succinate did not reduce BV-2 microglia-mediated neurotoxicity since NSC-34 neuronal viability was unaffected by diethyl succinate pre-treatment under all three stimulatory conditions (Figure 3C). 

### 3.4. Effects of Disodium Succinate on Phagocytic Activity and Mitochondrial Respiration

**Figure 4:**
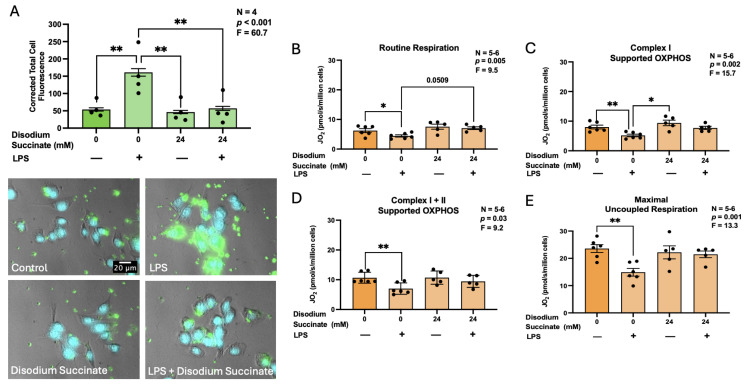
The effects of disodium succinate on the phagocytosis of fluorescent latex beads (**A**) by BV-2 murine microglia and mitochondrial respiration of human PBMCs (**B**–**E**). A: BV-2 cells were treated with disodium succinate (24 mM) or vehicle (PBS) for 3 h, followed by exposure to LPS (400 ng/mL) or its vehicle for 24 h, and then incubated with fluorescent latex beads for 1 h. Representative fluorescence images from the four experimental conditions are shown, illustrating latex bead uptake (green). Cell nuclei were counterstained with bisbenzimide (blue). Scale bar = 20 μm. Corrected total cell fluorescence was quantified by an investigator blinded to the experimental conditions and is presented as a bar graph. (**B**–**E**): PBMCs were treated with disodium succinate (24 mM), LPS (400 ng/mL), or both disodium succinate and LPS for 24 h. Mitochondrial respiration was determined using high-resolution respirometry. Routine respiration in intact cells (**B**) was determined prior to cell permeabilization (5 µg/mL digitonin) and addition of substrates for complex I (C; 2 mM malate, 5 mM pyruvate, 10 mM glutamate) and complex II ((**D**); 10 mM succinate) in the presence of saturating ADP (2.5 mM) to determine their OXPHOS capacity. Maximal uncoupled respiration (**E**) was determined by stepwise titrations of CCCP (0.05 µM steps). Data from four to six independent experiments completed on different days are shown as means ± SEM. While fluorescence was measured in 186–199 individual cells per experimental condition, only the averages from the four independent experiments are shown in (**A**). The displayed *p* and F values are calculated using randomized block one-way ANOVA. * *p* < 0.05 and ** *p* < 0.01 according to Tukey’s post hoc test.

To further elucidate the potential anti-inflammatory effects of extracellular succinate, we investigated its regulation of the phagocytic activity of BV-2 microglia, which is one of the essential immune functions of this cell type. While O’Callaghan et al. [33] report that intracellular succinate impairs bacterial phagocytosis in BMDMs, it remains unknown whether extracellular succinate exerts a similar effect in CNS-resident immune cells. To address this knowledge gap, we treated BV-2 murine microglia with disodium succinate in the presence or absence of LPS, a well-established inducer of microglial phagocytic activity [34]. Figure 4A demonstrates that pre-treatment with a non-toxic concentration (24 mM) of disodium succinate significantly reduced phagocytosis of latex beads by LPS-stimulated, but not unstimulated BV-2 cells. 

Reactive microglia not only exhibit enhanced phagocytic activity, but also undergo metabolic reprogramming characterized by decreased mitochondrial respiration and a shift toward glycolysis during their immune activation [35]. This metabolic transition may be necessitated, in part, by the high energetic demands associated with increased phagocytosis. Within the mitochondria, succinate functions as a key intermediate in the TCA cycle, contributing to OXPHOS-mediated ATP production, and as such participates in regulation of the cellular metabolism [12]. However, it remains unclear whether extracellular succinate can regulate mitochondrial respiration. To explore this possibility, we used human PBMCs as human microglia models [36] to measure changes in oxygen consumption under various modes of mitochondrial respiration, an established proxy for mitochondrial OXPHOS. Consistent with the ability of LPS to promote metabolic rewiring from oxidative metabolism towards glycolysis [37], we observed a global attenuation of all respiratory parameters (Figure 4B–D) after a 24 h exposure of human PBMCs to this immune stimulus. Disodium succinate rescued LPS-induced drop in respiratory capacity as reflected by increased routine (Figure 4B) and complex I-supported OXPHOS (Figure 4C). Similar patterns were apparent for complex I + II-supported OXPHOS (Figure 4D) and maximal uncoupled respiration (Figure 4E), although the effects of disodium succinate on these two parameters did not reach statistical significance. Disodium succinate on its own did not alter any of the respiratory parameters assessed compared to the cells not exposed to LPS or succinate (Figure 4B–E).

## 4. Discussion

In addition to its traditional role as a TCA cycle intermediate, succinate is emerging as an important intra- and extracellular signaling molecule. During an immune response, succinate can accumulate within the cytosol and the extracellular space, phenomena demonstrated in both peripheral and CNS cells [15,29,38]. In this study, we focused on the possible DAMP-like activity of extracellular succinate and investigated its ability to regulate select immune functions of microglia. One of the objectives of our research was to determine whether succinate, when acting extracellularly, had an overall pro- or anti-inflammatory impact on microglia. We also compared microglia-regulating effects of the cell membrane-impermeable succinate derivative to those of the cell-permeable one in order to identify location-dependent effects of this mediator. Finally, we assessed whether extracellularly acting succinate can modulate microglial metabolic activity by measuring mitochondrial respiration in human microglia model cells.

Initially, we investigated the effects of disodium succinate on microglia production of pro-inflammatory mediators TNF, IL-6, and NO. While inflammation in an acute sense may be beneficial, prolonged release of these molecules, for example, in many neurodegenerative disorders, can be toxic to neurons and other glial cells [39]. We stimulated BV-2 cells with LPS, IFN-γ, and their combination, and observed the previously reported synergistic effect of LPS and IFN-γ on cytokine production in these microglia cells [4,25]. Extracellular succinate at non-toxic concentrations downregulated TNF and IL-6 secretion by microglia stimulated with a combination of LPS plus IFN-γ but had no effect on cytokine secretion by unstimulated cells. This aligns with a study by Kirova et al. [21] demonstrating that injection of ethylmethylhydroxypyridine succinate decreases TNF and IL-1β expression in the brains of 18-month-old mice. However, our data differ from findings of Harber et al. [18], who report that disodium succinate does not alter secretion of TNF or IL-6 by BMDMs, a peripheral immune cell type more closely resembling primary microglia than the BV-2 cell line used in the present study. These differences suggest that regulation of cytokine secretion by extracellular succinate may depend on the specific characteristics of the cell type examined.

We also demonstrated, for the first time, the potential neuroprotective nature of extracellular disodium succinate wherein it reduced overall cytotoxicity of immune-stimulated microglia. Previously, succinate accumulation in murine brains has been associated with neuronal injury and increased excitability, leading to convulsive behaviour [40,41]. However, this neurotoxicity may be specific to succinate accumulation following the initiation of an immune response under adverse microenvironments, such as those involving hypoxia and inflammation [38,40]. In our study, we showed that pre-treatment of microglia with disodium succinate prior to immune stimulation mitigated their neurotoxic effects and rescued neuronal viability. Notably, disodium succinate had no effect on NO production by immune-stimulated microglia, which indicates that, by acting extracellularly, succinate regulates only select immune functions of microglia. 

The second focus of our study was to determine if the effect of succinate differs depending on whether it acts inside the cell or from outside by comparing the inhibitory activities of its diethyl and disodium derivatives. It should be noted that neither derivative was toxic to BV-2 cells at the concentrations used. In contrast, LPS and IFN-γ, alone or in combination, reduced BV-2 microglia viability, consistent with the findings of Qin et al. [42], who report LPS plus IFN-γ-induced apoptosis in RAW 264.7 murine macrophages. Wang et al. [20] have already shown that exposure of primary murine microglia to diethyl succinate decreases LPS-induced production of TNF. We extended these studies by using murine BV-2 microglia and directly comparing the effects of the disodium and diethyl forms of succinate on functional parameters that were responsive to the application of extracellular succinate. Our study revealed that the cell membrane-permeable diethyl succinate downregulated TNF and IL-6 production by LPS plus IFN-γ-stimulated BV-2 cells, similar to the actions of extracellular disodium succinate. Diethyl succinate might have been even more effective than disodium succinate, as it produced a higher percent inhibition of TNF secretion in LPS plus IFN-γ-stimulated microglia. Furthermore, diethyl succinate reduced LPS-induced TNF secretion, whereas disodium succinate only exhibited a trend toward significant inhibition under this experimental condition. However, unlike disodium succinate, diethyl succinate did not reduce neuronal death induced by immune-stimulated microglia, indicating that this mediator must act extracellularly to lower the overall neurotoxic activity of microglia. This likely reflects the multifactorial nature of microglia-induced neuronal damage, which is mediated by a mixture of inflammatory and cytotoxic factors beyond TNF and IL-6 (reviewed in [4]). Thus, while diethyl succinate effectively suppresses specific cytokines, disodium succinate provides broader extracellular modulation of microglial neurotoxicity, accounting for its protective effect on NSC-34 cell viability. It is important to note that the concentrations used in this study (up to 24 mM disodium succinate and 5 mM diethyl succinate) are consistent with prior in vitro studies of succinate signaling. Although higher than typical physiological levels, transient elevations of extracellular succinate have been reported in pathological contexts such as ischemia and CNS injury, suggesting potential pathophysiological relevance. Importantly, our preliminary experiments confirmed that these concentrations were non-toxic to the cell types examined and allowed us to probe mechanisms of succinate signaling.

A reason behind such location-dependent effects of succinate may be the activation of distinct signaling pathways. As mentioned previously, extracellular succinate can act as an agonist of SUCNR1, which is a G protein–coupled receptor expressed in a variety of tissues, including the kidney, small intestine, liver, heart, and immune cells [19]. Among CNS cell types, microglia are the primary cells expressing this receptor in both humans and mice [43,44]. Our digital PCR analysis demonstrated low levels of *Sucnr1* expression in unstimulated BV-2 murine microglia, which were significantly upregulated after 12 h of IFN-γ plus LPS stimulation. These data are consistent with a previous microarray analysis of G protein-coupled receptors expressed across multiple cell lines, which reported the presence of *Sucnr1* mRNA in BV-2 microglia, although expression levels did not reach statistical significance [45]. Together, these observations support the conclusion that the cellular responses to extracellular succinate observed in our study are mediated by this receptor. While these findings are consistent with the involvement of SUCNR1, our data do not directly establish receptor-dependent causality, and future studies using pharmacological inhibition or genetic manipulation of SUCNR1 will be necessary to conclusively determine the extent to which the observed extracellular succinate–mediated effects are SUCNR1-dependent. Consistent with this interpretation and complementary to our findings with disodium succinate, SUCNR1 activation has also been reported to reduce inflammatory responses in immune cells. For example, Keiran et al. [16] found that the succinate-SUCNR1 signaling caused primary murine macrophages to adopt an anti-inflammatory phenotype. Mechanistically, this study demonstrated that succinate–SUCNR1 signaling activates intracellular cyclic AMP-dependent pathways, including phosphorylation of protein kinase A (PKA) and the transcription factor cyclic AMP response element binding protein (CREB), leading to induction of the anti-inflammatory regulator KLF4 and increased expression of anti-inflammatory genes such as IL-10. Pharmacological inhibition of PKA or knockdown of Klf4 attenuated these succinate-induced transcriptional responses, supporting a functional role for the PKA–CREB–KLF4 signaling axis downstream of SUCNR1 activation [16]. Notably, these findings were obtained in peripheral macrophages, and it remains unclear whether similar intracellular signaling mechanisms operate in microglia. Future studies examining SUCNR1-dependent activation of the PKA–CREB pathway and its downstream transcriptional targets in microglia will be important to define the intracellular signaling cascades mediating succinate-induced modulation of microglial function. In a separate study, implant of SUCNR1 loss-of-function neural stem cells in mice with chronic experimental autoimmune encephalomyelitis (EAE) led to an impaired ability to ameliorate chronic neuroinflammation further indicating possible inflammation-resolving outcomes of succinate-SUCNR1 interaction [46].

In contrast, succinate acting intracellularly may operate through SUCNR1-independent mechanisms. Evidence for this comes from Wang et al. [20] who report that diethyl succinate decreases TNF and ROS production in both SUCNR1 knockout and control murine microglia. Similarly, Harber et al. [18] find that this cell-permeable succinate derivative inhibits LPS (+/−IFN-γ)-induced expression of pro-inflammatory mediators and cell surface markers to an equivalent extent in wild-type and SUCNR1-deficient BMDMs. Therefore, cytosolic succinate is likely to influence cellular functions by engaging other, SUCNR1-independent mechanisms. In addition, it cannot be excluded that intracellular hydrolysis of diethyl succinate to succinate leads to back-transport of succinate into the extracellular space [16,17], where it could interact with SUCNR1. The SUCNR1-independent mechanisms include, for example, the natural accumulation of succinate in macrophages under hypoxic conditions, which stabilizes hypoxia-inducible-factor-1α (HIF-1α) and influences ROS through reverse electron transport [19,47]. Succinate located intracellularly can also act as a substrate for succinyl-CoA to facilitate lysine succinylation, a post-translational modification used to regulate metabolic enzyme activities in mitochondria [48]. Interestingly, a decrease in succinylation of vitamin D receptor in BV-2 murine microglia upregulates expression of IL-1β, IL-2, IL-6, IL-10, IL-12, monocyte chemoattractant protein (MCP)-1, and TNF, implicating this biochemical process in the regulation of inflammatory mechanisms [49]. Nevertheless, additional studies are required to elucidate the precise molecular mechanisms by which succinate exerts its effects in extracellular versus intracellular contexts. 

To further understand the immunomodulatory effects of extracellular succinate, we explored the impact of disodium succinate on the phagocytic activity of BV-2 microglia. As professional phagocytes, microglia often aid in the clearance of cell debris or damaged neurons to maintain tissue homeostasis and immune tolerance [50,51]. Such examples of phagocytosis are beneficial and should not be altered. However, under certain pathological conditions, phagocytic activity of microglia can become exacerbated, leading to the destruction of viable neurons and contributing to neurodegeneration [52,53]. Here we show that disodium succinate mitigated LPS-induced upregulation of phagocytosis of fluorescent latex beads by BV-2 microglial cells. While latex bead-based assays are widely used due to their sensitivity and quantitative reproducibility, inert particles do not fully recapitulate the complexity of physiological phagocytic substrates. In particular, bead uptake may not accurately reflect receptor-specific interactions involved in the clearance of myelin, synaptic elements, or cellular debris in vivo [54]. Therefore, our findings should be interpreted as reflecting modulation of general phagocytic capacity rather than specific physiological pathways. Future studies using more biologically relevant substrates, such as synaptosomes, myelin debris, or neuronal cells, will be important to validate these observations. Nevertheless, it is notable that disodium succinate did not affect the phagocytic activity of unstimulated cells, allowing for normal levels of phagocytosis in the absence of immune stimuli. This selective inhibition towards only immune-stimulated cells is advantageous in the context of overactive microglia and thus, considered anti-inflammatory. It also aligns with our other findings demonstrating that extracellular succinate can aid in regulation of microglia immune functions. 

The role of succinate in inflammation has been debated, and limited CNS research has left its neuroimmune functions incompletely understood. Our findings demonstrate that extracellular succinate acts predominantly as an anti-inflammatory signal in microglia by reducing pro-inflammatory cytokine secretion, neurotoxicity, and excessive phagocytosis without disrupting basal microglial activity. This is consistent with Wang et al. [20], who reported anti-inflammatory effects of intracellular succinate in primary murine microglia, including reduced LPS-induced ROS production and decreased secretion of TNF and IL-1β. However, our data suggest that extracellular succinate may engage distinct signaling pathways, potentially involving SUCNR1, which requires further investigation.

Our third objective was to determine whether extracellular succinate modulates cellular metabolism, specifically mitochondrial respiration. While inflammatory activation of BV-2 cells, as well as microglia broadly, is linked to increased glycolysis [35,55], we used human PBMCs for these experiments to better approximate human microglial metabolic responses instead of using murine cells. We demonstrated that, similar to past studies with other mononuclear cells [56,57], LPS treatment resulted in a decrease in PBMC oxygen consumption, reflecting a reduction in ATP production via OXPHOS. However, given the heterogenous nature of PBMCs that include both monocytes and lymphocytes with distinct metabolic properties, future work should measure mitochondrial bioenergetics in specific human leukocyte subpopulations or human microglia. Nonetheless, decrease in oxygen consumption is typical for immune cells, including microglia, transitioning to pro-inflammatory phenotypes where the cells switch from OXPHOS to glycolysis to ensure more rapid energy production [58]. Interestingly, treatment with extracellular disodium succinate rescued LPS-induced downregulation of complex I-supported OXPHOS in PBMCs and a trend toward a similar effect was observed for routine (basal) respiration. Although non-significant, the pattern of response—namely, the rescue of LPS-induced downregulation of respiration—was consistent across complex I- and II-supported OXPHOS and maximal uncoupled respiration. Previously, Vasilopoulou et al. [30] have shown that deficits in maximal respiration and spare respiratory capacity observed in human iPSC-derived microglia, in which AD-linked gene variants were generated, is mitigated by disodium succinate at 10 mM, consistent with our findings demonstrating its effectiveness at 24 mM. Additional research will be necessary to clarify the mechanism behind this effect, as the current dataset is limited to PBMCs from five donors (3 males, 2 females). Given the known sexual dimorphism and intersubject variability in human immune responses [59], future work should use larger sample sizes with balanced numbers of males and females to explore sex-based differences in immune responses to succinate.

The ability of extracellular succinate to increase OXPHOS may also explain its effects on microglia phagocytic activity. Because phagocytosis is a high-energy process, it can be linked to changes in cellular metabolism. For instance, immune-stimulated microglia undergo metabolic reprogramming towards glycolysis, a faster albeit less efficient method to generate ATP [60]. Such a switch, even in the presence of sufficient oxygen, is similar to the Warburg effect in tumor cells and can help meet the energy demands of actively phagocytosing cells [61]. By increasing OXPHOS, disodium succinate may have an opposing effect whereby glycolysis is downregulated. This could limit the rate of cellular energy production, and subsequently, may slow down phagocytosis, explaining the decrease in this immune function that was observed after treatment with disodium succinate. Therefore, future studies could measure glycolytic flux, assessed as extracellular acidification rate (ECAR), to determine whether succinate-mediated rescue of OXPHOS is accompanied by reciprocal changes in glycolysis. Nevertheless, our findings highlight a potential mechanistic link between extracellular succinate signaling and the metabolic regulation of microglial function. 

## 5. Conclusions

Our data demonstrate that extracellular succinate may function in the CNS as a novel immunomodulatory DAMP, exerting predominantly anti-inflammatory effects on microglia. Specifically, succinate acting in the extracellular space reduces microglial secretion of pro-inflammatory cytokines, mitigates microglia-mediated neurotoxicity, and selectively downregulates excessive phagocytic activity in immune-stimulated microglia. Importantly, several of these effects are mediated by extracellular—but not intracellular—succinate, underscoring that the location of this metabolite within or outside the cell is a key factor in determining its effects on microglia. Additionally, the observed modulation of microglial metabolism by restoring mitochondrial respiration in immune-stimulated cells may represent a mechanistic link between extracellular succinate signaling and regulation of phagocytic activity. As such, further work to fully elucidate the molecular mechanisms engaged by extracellular succinate in microglia is warranted. 

Collectively, this study expands our understanding of succinate as an immunometabolic regulator in the CNS, revealing its capacity to modulate detrimental microglial activation known to contribute to neuroinflammation and neurodegeneration. Due to its selective anti-inflammatory and metabolic effects, extracellular succinate may represent a promising therapeutic approach for restoring immune homeostasis in neurological diseases. Future investigations using human microglia and advanced in vitro models, such as brain organoids, will be essential for exploring the therapeutic potential of targeting succinate signaling in neuroimmune disorders.

## Data Availability

The original contributions presented in this study are included in the article/Supplementary Materials. Further inquiries can be directed to the corresponding author(s).

## Supplementary Materials

The following supporting information can be downloaded at: https://www.mdpi.com/article/10.3390/biom16030407/s1. Supplementary Figure S1: The effects of disodium succinate on the secretion of TNF (A), IL-6 (B), and NO (C) by unstimulated BV-2 murine microglia and their viability (D); Figure S2: The effects of diethyl succinate on the secretion of TNF (A) and IL-6 (B) by unstimulated BV-2 murine microglia and their viability (C); Figure S3: Effects of IFN-γ, LPS, disodium succinate, and diethyl succinate on the viability of NSC-34 murine neuron-like cells.; Figure S4: Raw digital PCR data from the QuantStudio Absolute Q Digital PCR System showing the FAM fluorescence signal per droplet for each sample in the Actb assay (top panel) and Sucnr1 assay (bottom panel).
