# Supplementary material for: Extracellular Succinate Modulates Neuroimmune Responses in a Murine Microglial Cell Line
Source: Biomolecules. 2026 Mar 10;16(3):407. doi: 10.3390/biom16030407 (PMC13024544; doi:10.3390/biom16030407)
Supplement: Supplementary file 1 [file biomolecules-16-00407-s001.zip › biomolecules-4161744-supplementary.pdf]

Extracellular Succinate Modulates Neuroimmune Responses in a Murine Microglial Cell Line

Samantha C.Y. Yudin [1], Kimberly Day [2], Erica Y. Scott [1], Meha N. Patel [1], Hashim Islam [2,*], Andis Klegeris [1,*]

Supplementary Materials

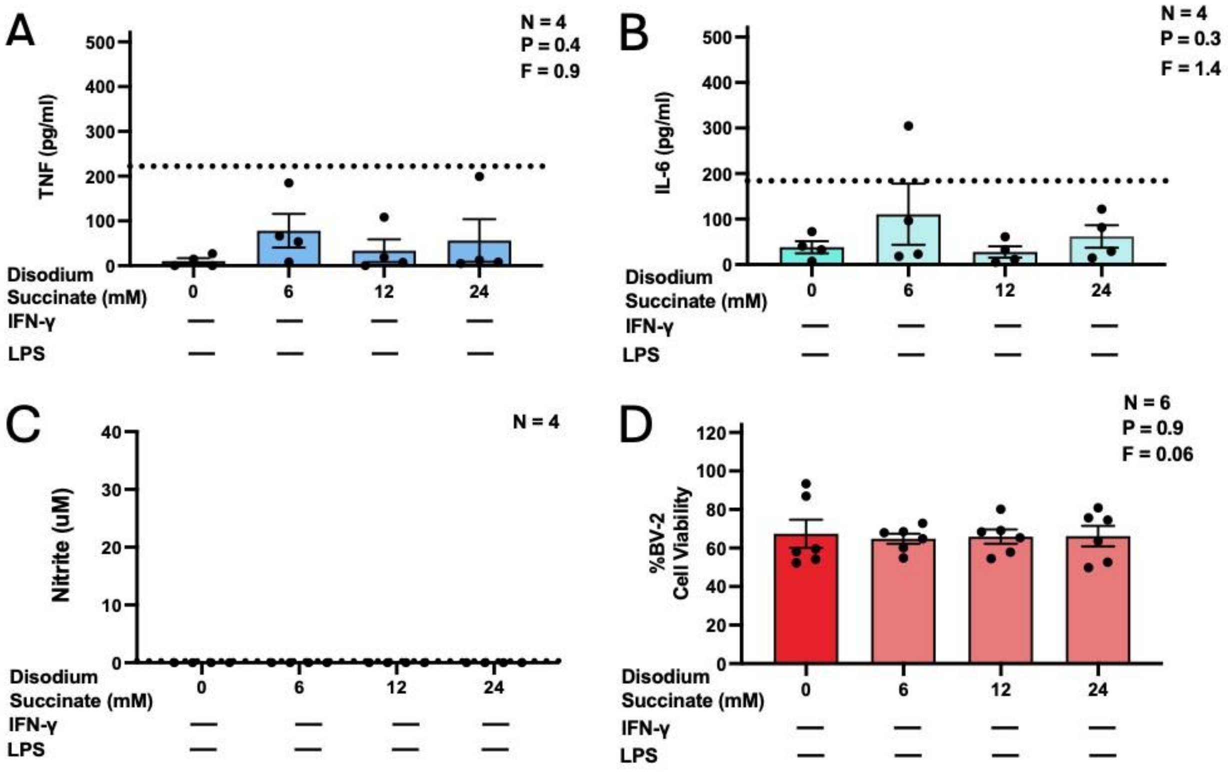

Figure S1. The effects of disodium succinate on the secretion of TNF (A), IL-6 (B), and NO (C) by unstimulated BV-2 murine microglia and their viability (D). Cells were treated with disodium succinate (0-24 mM) for 3 h. Following a 24 h incubation period, concentrations of cytokines and nitrite in cell-free supernatants were measured by ELISAs and the Griess assay, respectively. Viability of the BV-2 cells was measured by the MTT assay. Data from four (A-C) or six (D) independent experiments completed on different days are shown as means ± SEM. The displayed P and F values are calculated using randomized block one-way ANOVA, and the detection limits of the ELISAs and Griess assay are indicated by a dotted line.

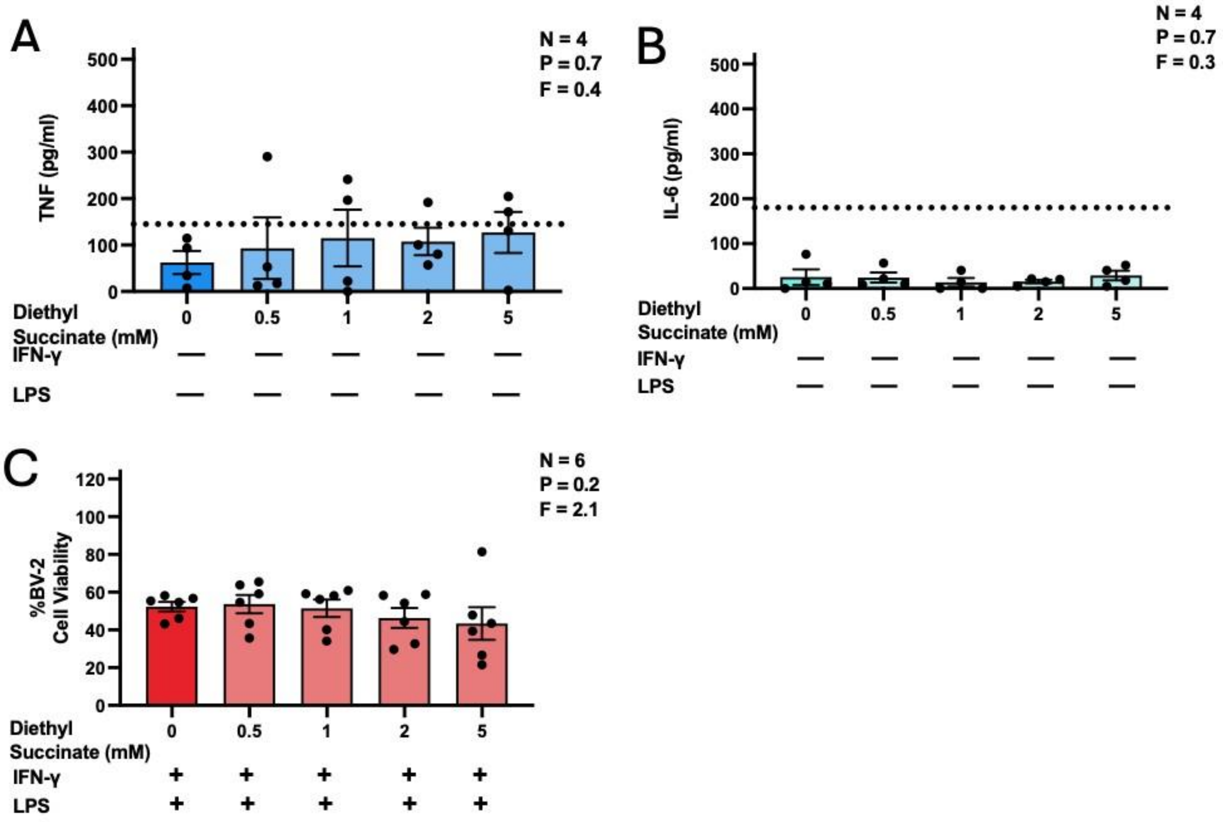

Figure S2. The effects of diethyl succinate on the secretion of TNF (A) and IL-6 (B) by unstimulated BV-2 murine microglia and their viability (C). Cells were treated with diethyl succinate (0-5 mM) for 3 h. Following a 24 h incubation period, concentrations of IL-6 were measured by an ELISA and viability of the BV-2 cells was measured by the MTT assay. Data from four (A, B) or six (C) independent experiments completed on different days are shown as means ± SEM. The displayed P and F values are calculated using randomized block one-way ANOVA, and the detection limit of the ELISA is indicated by a dotted line.

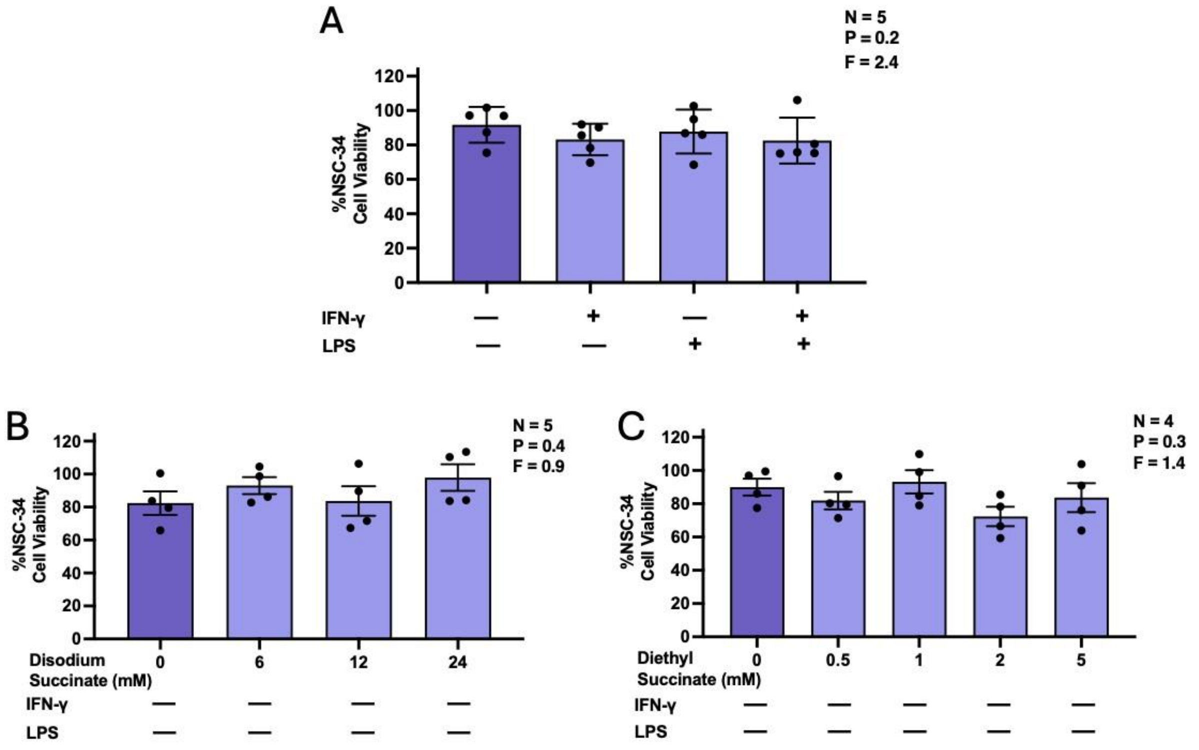

Figure S3. Effects of IFN-γ, LPS, disodium succinate, and diethyl succinate on the viability of NSC-34 murine neuron-like cells. NSC-34 cells were treated with IFN-γ (30 ng/mL), LPS (400 ng/mL), or their combination (A), disodium succinate (0-24 mM) (B), or diethyl succinate (0-5 mM) (C). These concentrations correspond to those used to treat BV-2 murine microglia (see Figures 2 and 3). Following a 72 h incubation period, the viability of NSC-34 cells was quantified using the MTT assay. Data from five independent experiments completed on different days are shown as means ± SEM. The displayed P and F values are calculated using randomized block one-way ANOVA.

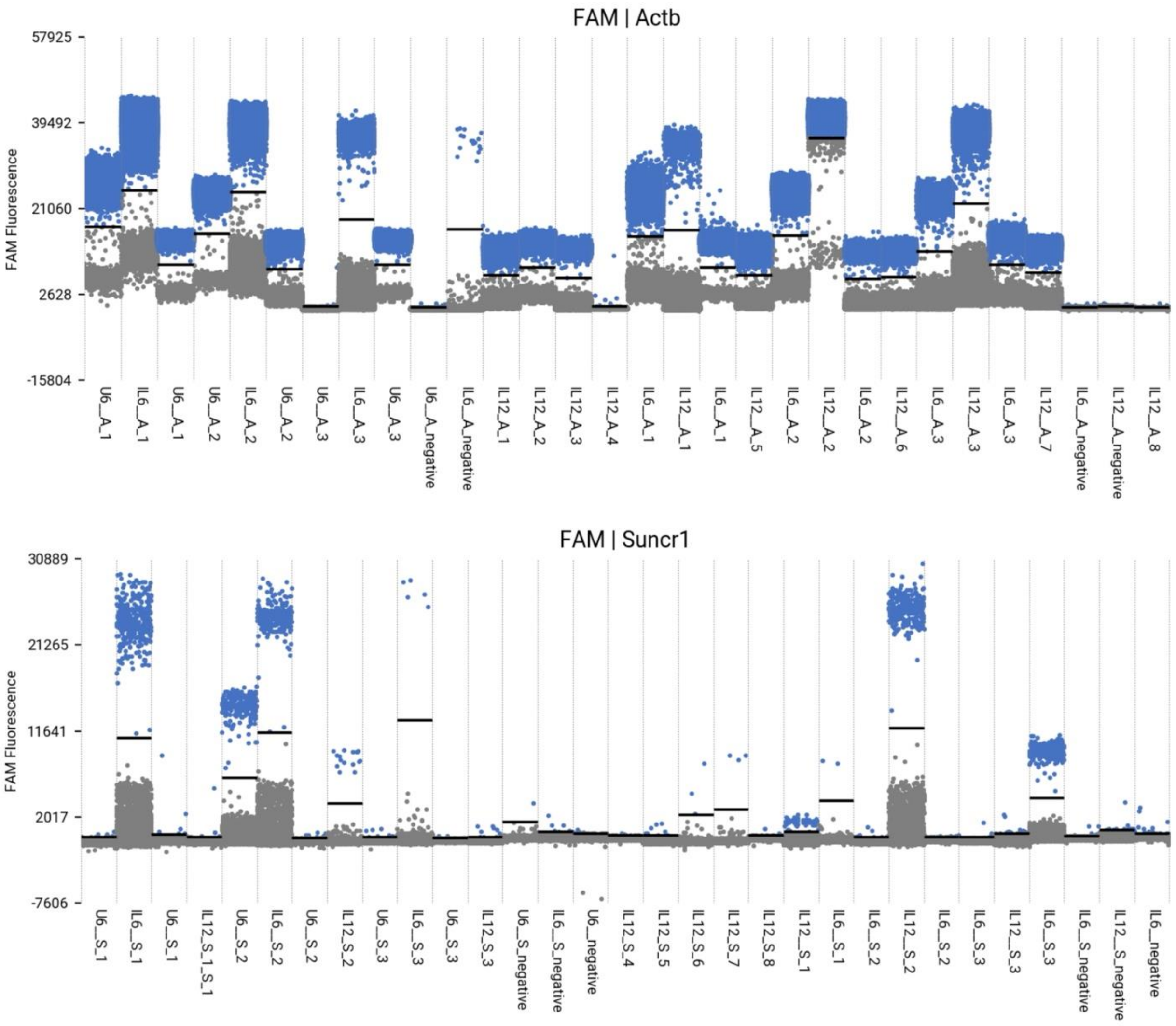

Figure S4. Raw digital PCR data from the QuantStudio Absolute Q Digital PCR System showing the FAM fluorescence signal per droplet for each sample in the *Actb* assay (top panel) and *Sucnr1* assay (bottom panel). In these plots, blue dots indicate positive calls, and grey dots indicate negative calls (y-axis). The BV-2 murine microglia samples correspond to those shown in Figure 2C and are labeled as follows on the x-axis: U6, untreated cells for 6 h; IL6, cells treated with IFN-γ and LPS for 6 h; and IL12, cells treated with IFN- γ and LPS for 12 h.
